# Performance of the Global Diet Quality Score with Nutrition and Health Outcomes in Mexico with 24-h Recall and FFQ Data

**DOI:** 10.1093/jn/nxab202

**Published:** 2021-10-23

**Authors:** Analí Castellanos-Gutiérrez, Sonia Rodríguez-Ramírez, Sabri Bromage, Teresa T Fung, Yanping Li, Shilpa N Bhupathiraju, Megan Deitchler, Walter Willett, Carolina Batis

**Affiliations:** Health and Nutrition Research Center, National Institute of Public Health, Cuernavaca, Mexico; Health and Nutrition Research Center, National Institute of Public Health, Cuernavaca, Mexico; Harvard T.H. Chan School of Public Health, Harvard University, Boston, MA, USA; Harvard T.H. Chan School of Public Health, Harvard University, Boston, MA, USA; Department of Nutrition, Simmons University, Boston, MA, USA; Harvard T.H. Chan School of Public Health, Harvard University, Boston, MA, USA; Harvard T.H. Chan School of Public Health, Harvard University, Boston, MA, USA; Intake – Center for Dietary Assessment, FHI Solutions, Washington, DC, USA; Harvard T.H. Chan School of Public Health, Harvard University, Boston, MA, USA; CONACYT—Health and Nutrition Research Center, National Institute of Public Health, Cuernavaca, Mexico

**Keywords:** GDQS, diet quality, nutrient intake, nutrient adequacy, noncommunicable disease, Mexican women, ENSANUT, 24-h recall, FFQ

## Abstract

**Background:**

The Global Diet Quality Score (GDQS) is intended as a simple global diet quality metric feasible in low- and middle-income countries facing the double burden of malnutrition.

**Objective:**

The aim of this study was to evaluate the performance of the GDQS with markers of nutrient adequacy and chronic disease in nonpregnant nonlactating (NPNL) Mexican women of reproductive age and to compare it with the Alternate Healthy Eating Index-2010 (AHEI-2010) and the Minimum Dietary Diversity for Women (MDD-W).

**Methods:**

We included NPNL women aged 15 to 49 y from the Mexican National Health and Nutrition Surveys (2012 and 2016) with 24-h recall (*n* = 2542) or a FFQ (*n* = 4975) (separate samples). We evaluated the correlation of the GDQS with the energy-adjusted intake of several nutrients and evaluated its association with health parameters using covariate-adjusted linear regression models.

**Results:**

The GDQS was positively correlated with the intake of calcium, folate, iron, vitamin A, vitamin B-12, zinc, fiber, protein, and total fat (rho = 0.09 to 0.38, *P* < 0.05) and was inversely correlated with the intake of added sugar (rho = −0.37 and −0.38, *P* < 0.05) using both instruments, and with total fat, SFA, and MUFA only with 24-h recall data (rho = −0.06 to −0.16, *P* < 0.05). The GDQS was inversely associated with serum ferritin, BMI, waist circumference, and serum total and LDL cholesterol using FFQ data (*P* < 0.05), and was positively associated with serum folate using 24-h recall data (*P* < 0.05). Similar correlations and associations were observed with the MDD-W (only with micronutrients) and the AHEI-2010 (only with chronic disease-related nutrients and health markers).

**Conclusions:**

In comparison to other diet metrics, the GDQS can capture both dimensions of nutrient adequacy and health markers related to the risk of chronic disease. The performance of the GDQS was satisfactory with either 24-h recall or FFQ.

## Introduction

Poor diet quality is one of the main drivers of noncommunicable disease (NCD) morbidity and mortality globally (1). In 2017, one-fifth of all deaths in adults and 15% of disability-adjusted life years (DALYs) were attributed to dietary risk factors, mainly high intake of sodium, low intake of whole grains, and low intake of fruits. The double burden of malnutrition, which refers to both undernutrition and overweight/obesity, continues to be a significant problem in many countries, in particular in low- and middle-income countries (LMICs) (2). Consequently, the importance of adequately measuring diet quality at the population level is apparent and has been met with increasing efforts to develop low-burden diet quality instruments that are feasible in LMICs where resources for data collection are limited.

The nutritional transition that has occurred in Mexico over the last decades has resulted in the steep rise of overweight/obesity and its associated NCDs (3). In 2016, two-thirds of the adult population was considered overweight or obese (4), 1 in 10 adults was diagnosed as having diabetes (5), and a quarter had high blood pressure (6). At the same time, the diet of the population is not providing enough nutrients to fulfill individual requirements, resulting in a high prevalence of inadequate intake of micronutrients across all segments of the Mexican population (7).

The Global Diet Quality Score (GDQS) was developed to address the need for a simple metric that could capture both dimensions of diet quality related to risk of NCDs and nutrient adequacy at the population level and that can be tabulated without the need for food composition tables which may not be available in many LMICs. Moreover, existing metrics of diet quality were mostly developed using data from high-income countries (HICs) and therefore, it is unclear whether they can adequately measure diet quality dimensions in LMICs given their distinct dietary patterns (8). The GDQS is intended to be used at a population level using dietary data obtained with a 24-h dietary recall or an FFQ and therefore, it is important to understand the advantages and disadvantages provided by both methods for this purpose. The 24-h recall captures detailed information about food intake and is less prone to systematic error compared with other methods, and the FFQ is a more affordable tool designed to capture long-term intake but more susceptible to bias and less reliable for estimating absolute intake of foods and nutrients (9–11). Alternatively, data can be collected using the GDQS app, an electronic tool developed to provide a simple and low-burden instrument for data collection when a 24-h recall or FFQ are not feasible (12).

The GDQS was developed with an initial focus on nonpregnant nonlactating (NPNL) women of reproductive age for their condition as a high-priority group for targeting nutrition interventions given the relevance of their nutritional status for themselves and their offspring.

As part of the research initiative to develop a novel metric to measure diet quality that would be appropriate to use in LMICs, the objective of this study was to evaluate the performance of the GDQS with markers of nutrient adequacy and NCDs in NPNL Mexican women of reproductive age. We used dietary data from the Mexican National Health and Nutrition Survey (ENSANUT) from 2012 and 2016 obtained with 2 different instruments (24-h recall and FFQ). Additionally, we compared the performance of the GDQS with the Alternate Healthy Eating Index-2010 (AHEI-2010), a widely used metric associated with the risk of chronic disease (13), and with the Minimum Dietary Diversity for Women (MDD-W), a population-level food group diversity indicator that serves as a proxy measure of micronutrient adequacy in women (14).

## Methods

### ENSANUT

We analyzed data from NPNL women aged 15 to 49 y from the ENSANUT surveys from 2012 and 2016. Information was collected by face-to-face interviews carried out between October 2011 and May 2012 for ENSANUT 2012 and between May and October 2016 for ENSANUT 2016. Informed consent was obtained from participants aged ≥18 y and from the parent or guardian of participants aged <18 y. The survey protocol was approved by the Ethics Committee of the National Institute of Public Health (INSP).

### 24-h recall

A random subsample of ∼11% (*n* = 10,886) of the total respondents of ENSANUT 2012 and ∼15% (*n* = 4341) of ENSANUT 2016 was selected to provide dietary information using a 5-step multiple-pass 24-h dietary recall method developed by the USDA and adapted to Mexican context. The detailed methodology for data collection has been described elsewhere (7, 15). Briefly, participants provided detailed information about all the foods consumed on the day prior to the interview. Information about food quantity was provided in household measures or food weight. Interviews were conducted by previously trained and standardized personnel using an automated software that follows a series of iterative steps to aid memory and minimize underreporting. A second 24-h recall was administered to a randomly selected subsample of ∼9% of the participants that responded to the first recall. Both interviews were administered on nonconsecutive days between Monday and Sunday.

### FFQ

In a separate subsample of the ENSANUT 2012 and 2016, dietary information was collected using a semiquantitative FFQ that inquires about the intake over the past 7 d. The FFQ includes 140 food items with the following response options: never, 1 d/wk, 2–4 d/wk, 5–6 d/wk, 7 d/wk; and a times per day frequency of: 1/d, 2–3/d, 4–5/d, and 6/d. Respondents also selected a portion size from predefined standard sizes and then indicated the number of portions consumed. We used the information on frequency, portion size, and number of portions to compute intake per day (g/d) for each item.

### The GDQS

The GDQS is comprised of 25 food groups divided into 3 or 4 intake categories defined by pre-established cutoffs (g/d) and are scored according to their health implications (16). Healthy food groups (16 out of 25) were positively scored, meaning that higher scores were assigned to higher intakes. Unhealthy food groups (9 out of 25) were negatively scored, with the exception of high fat dairy and red meat that received higher scores with moderate intake and lower scores with very low or very high intakes. The GDQS ranges from 0 to 49 points and a higher score indicates higher dietary quality. The GDQS Positive submetric (GDQS+) is composed from the added score of the healthy food groups and ranges from 0 to 32 points, and the GDQS Negative submetric (GDQS–) is composed from the added score of the unhealthy food groups and ranges from 0 to 17 points.

We classified the foods reported in the first 24-h recall and in the FFQ to their corresponding GDQS food group. The mixed dishes included in the 24-h recall were disaggregated into individual foods specified by the respondent or following a standard recipe when the participant could not provide this information. The food items included in the FFQ that were complex mixed dishes were disaggregated into individual foods following a standard recipe and each ingredient was categorized in the corresponding GDQS food group. Industrialized dairy drinks with sugar were counted in the *dairy* group and home-made dairy-based drinks were disaggregated with only the milk products included in the *dairy* groups and the rest of the ingredients in their corresponding group. The foods in the group of *purchased deep fried foods* were “double-counted,” meaning that they were also included in the corresponding group according to their characteristics, i.e. “French fries” were included in deep fried foods and in white roots and tubers. Because there was not enough information to distinguish when foods were purchased or prepared at home, the *purchased deep fried foods* included single foods and mixed dishes that are typically acquired outside of home. We estimated each participant's daily intake (g/d) of every food group and assigned the point values that corresponded to their level of intake to compute the GDQS, GDQS+, and GDQS−. A more detailed description for the tabulation of the GDQS is described elsewhere in this supplement (16).

### Comparison metrics

We compared the performance of the GDQS with the AHEI-2010 and the MDD-W. The AHEI-2010 is a diet quality index based on 11 foods and nutrients that are predictive of chronic disease risk (13). We estimated daily intake for each component except for alcohol intake and assigned the corresponding score ranging from 0 to 10 points to the remaining 10 components, with a maximum total score of 100 points. A higher AHEI-2010 indicates higher diet quality and is associated with lower disease risk.

To estimate the MDD-W, we estimated daily intake (g/d) of 10 predefined food groups (14) and assigned a value of 1 point when intake was at or above 15 g/d and zero points otherwise. The MDD-W was developed to be used as a dichotomous indicator for dietary diversity using a cutoff of ≥5 food groups consumed the previous day to serve as a proxy for micronutrient adequacy at the population level. For this analysis, we used the values for the underlying dietary diversity score obtained by adding the points for the 10 food groups, with a possible range of values from 0 to 10 points. A higher score reflects higher dietary diversity and a better likelihood of micronutrient adequacy. A more detailed description of the estimation of the AHEI-2010 and the MDD-W can be found elsewhere in this supplement (16).

### Health parameters

Information on weight, height, and waist circumference (WC) measurements were obtained by trained and standardized personnel using conventional and internationally accepted protocols (17, 18).

To obtain measures of serum biomarkers, a venous blood sample was obtained after a minimum 8-h fast and was centrifuged in situ at 3000 × *g* for 20 min and stored at −70°C until assay at the laboratory of the Center for Nutrition and Health Research at INSP, Cuernavaca, Mexico.

Serum ferritin and folate concentrations were measured by chemiluminescent microparticle immunoassay technology in an autoanalyzer (ARCHITECT i 2000). Serum ferritin values were adjusted for the concentration of C-reactive protein following the Thurnham equation (19). Serum glucose was measured by an automatized glucose oxidase method using SynchronX equipment and insulin by chemiluminescence using Access2 equipment. Blood lipid determinations were obtained using an automatic immunoanalyzer (Architect C18200), triglycerides by lipase hydrolysis, total cholesterol by enzymatic digestion and oxidation, and HDL cholesterol by a direct enzymatic colorimetric method. LDL cholesterol was estimated according to the Friedewald equation (20).

Metabolic syndrome (MetS) was evaluated by estimating the number of risk factors (out of 5) defined by the National Cholesterol Education Program (NCEP) Adult Treatment Panel III (ATP III) (21) that were present for each woman. Risk factors included WC ≥80 cm, high blood pressure (known diagnosis or under pharmacological treatment), hypertriglyceridemia (≥150 mg/dL or under pharmacological treatment), low HDL cholesterol (<50 mg/dL or under pharmacological treatment), and hyperglycemia (≥100 mg/dL or under pharmacological treatment).

### Sociodemographic information

We obtained data on age, socioeconomic status (SES), and area of residence (urban/rural) from the ENSANUT 2012 and 2016 databases. SES is measured in ENSANUT with an index constructed using a first component analysis based on household information that included the type of construction materials, number of rooms used for sleeping, water supply, car ownership, and number of household goods and electrical appliances, and was divided into tertiles to categorize low, medium, and high SES. Urban areas were defined as locations with ≥2500 inhabitants and rural areas were those with <2500 inhabitants per basic geostatistical areas.

### Nutrient adequacy

We estimated individual daily intake of calcium, fiber, folate, iron, monounsaturated fatty acid (MUFA), protein, polyunsaturated fatty acid (PUFA), protein, saturated fatty acid (SFA), total fat, vitamin A, vitamin B-12, and zinc, as well as energy intake using the food composition tables compiled by INSP (22). The intake of added sugars was calculated following a 5-step algorithm that has been described elsewhere (23). We estimated usual nutrient intake and usual energy intake for the 24-h recall data obtaining the best linear unbiased predictors (BLUPs) following the Iowa State University (ISU) method using the Software for Intake Distribution Estimation PC-Side v.1.02 (24) and used the BLUPs to obtain energy-adjusted estimates of nutrient intake following the residual method (25).

For the FFQ data, we considered as adequate intakes those above the estimated average requirement (EAR) established by the U.S. Institute of Medicine DRIs for calcium, folate, vitamin A, and vitamin B-12, and above the adequate intake (AI) for fiber (26). The EAR for zinc was based on the recommendation by the International Zinc Nutrition Consultative Group (IZiNCG) for a bioavailability of 25% (27). For the 24-h recall data, nutrient adequacy for all nutrients except for iron was based on the probability of adequacy for the usual nutrient intake based on a normal distribution with a mean = EAR and SD = CV(EAR). Iron adequacy was evaluated following a full-probability approach based on requirement distributions adjusted to an assumed iron bioavailability of 10% (28).

Overall nutrient adequacy was evaluated based on the intake of calcium, fiber, folate, iron, protein, vitamin A, vitamin B-12, and zinc. For the FFQ data, the value for overall nutrient adequacy is the sum of nutrients with adequate intake and for the 24-h recall data, the value is the nutrients’ mean probability of adequacy (MPA).

### Statistical analysis

We estimated Spearman's correlation coefficients to evaluate the relation between the diet quality metrics and the energy-adjusted intake of nutrients. The association between the diet quality metrics and health outcomes was assessed using linear regression models with the *z*-score values of the metrics as predictors and adjusted by age, SES, and urban/rural area. Additionally, we estimated separate covariate-adjusted models to test for interaction between age groups (15–29 y, 30–39 y, and 40–49 y) and the GDQS, the AHEI-2010, and the MDD-W for its association with all health parameters and we present the predicted difference [β (95% CI)] in each group. An interaction was considered significant with a *P* value < 0.10. For all other analyses, estimates were considered significant with a *P* value < 0.05. To account for the possibility of overstating statistical significance in the correlation and regression analyses that include multiple comparisons, we have additionally indicated when estimations have a *P* value < 0.001. All correlations and regression models were conducted in STATA v. 14.0 (StataCorp).

## Results

We present the characteristics of the study sample and the mean values of the diet quality metrics across subpopulations in **Table 1** as well as the mean values of the individual components of the GDQS in **Supplemental Table 1**. The study sample was comprised of 2542 NPNL women (1655 from ENSANUT 2012 and 887 from ENSANUT 2016) with information from a 24-h recall and 4975 NPNL women (1737 from ENSANUT 2012 and 3248 from ENSANUT 2016) with information from an FFQ, between the ages of 15 and 49 y. The mean age was 30.7 ± 9.7 y for women with FFQ data and 28.0 ± 10.9 y for women with 24-h recall data. For both data sets, the GDQS increased with age and was slightly higher in the rural population compared with the urban population (*P* < 0.05). Across SES categories, the GDQS mean value was significantly higher in women with a low SES compared with medium and high SES for the 24-h recall data set, as opposed to the FFQ data set, with a higher GDQS in women in the high SES category. For the 24-h recall data, the GDQS+ and the GDQS− submetrics followed a pattern similar to the GDQS across subpopulations; whereas for the FFQ data, the submetrics followed opposite tendencies in regards to area of residence and SES, with a lower GDQS+ among rural women compared with urban women and increasing with SES, whilst the opposite was true for the GDQS−. The AHEI-2010 and MDD-W were also higher in older women compared with their younger counterparts. The AHEI-2010 was distributed in the same way as the GDQS− across urban/rural area and SES for both data sets, and the MDD-W followed the same distribution as the GDQS+ only for the FFQ data, whereas for the 24-h recall data the MDD-W increased with SES but did not differ between urban/rural area.

**TABLE 1:** Characteristics of the study population and distribution of the diet quality metrics^1^

	24-h recall						FFQ					
	*n* (%)	GDQS	GDQS+	GDQS−	AHEI-2010	MDD-W	*n* (%)	GDQS	GDQS+	GDQS−	AHEI-2010	MDD-W
All	2542 (100)	16.4 ± 4.0	6.4 ± 3.4	9.9 ± 2.5	44.3 ± 10.9	4.3 ± 1.5	4975 (100)	20.1 ± 3.8	11.1 ± 3.8	9.0 ± 2.4	52.8 ± 8.7	6.7 ± 1.5
Age												
15–29 y (Ref.)	1411 (55.5)	15.8 ± 4.1	6.2 ± 3.3	9.7 ± 2.6	43.0 ± 11.0	4.2 ± 1.5	1868 (37.5)	19.3 ± 3.7	10.6 ± 3.7	8.6 ± 2.5	51.2 ± 8.8	6.6 ± 1.5
30–39 y	621 (24.4)	16.8 ± 3.8^2^	6.6 ± 3.2^2^	10.2 ± 2.3^2^	45.3 ± 10.3^2^	4.3 ± 1.5^2^	1661 (33.4)	20.3 ± 3.7^2^	11.3 ± 3.7^2^	9.1 ± 2.4^2^	53.1 ± 8.5^2^	6.8 ± 1.5^2^
40–49 y	510 (20.1)	17.4 ± 3.9^2^	7.0 ± 3.6^2^	10.4 ± 2.4^2^	46.4 ± 10.7^2^	4.4 ± 1.6^2^	1446 (29.1)	21.1 ± 3.9^2^	11.6 ± 4.0^2^	9.5 ± 2.3^2^	54.4 ± 8.4^2^	6.8 ± 1.5^2^
Area												
Rural (Ref.)	1030 (40.5)	16.9 ± 3.8	6.7 ± 3.1	10.2 ± 2.3	46.8 ± 10.6	4.2 ± 1.5	2209 (44.4)	20.3 ± 3.7	10.9 ± 3.7	9.4 ± 2.4	54.1 ± 8.4	6.6 ± 1.6
Urban	1512 (59.5)	16.0 ± 4.1^2^	6.3 ± 3.5^2^	9.7 ± 2.6^2^	42.5 ± 10.7^2^	4.3 ± 1.5	2766 (55.6)	20.0 ± 3.9^2^	11.3 ± 3.9^2^	8.8 ± 2.4^2^	51.7 ± 8.8^2^	6.9 ± 1.4^2^
SES												
Low (Ref.)	833 (32.8)	16.8 ± 3.9	6.5 ± 3.1	10.3 ± 2.3	46.9 ± 11.0	4.1 ± 1.5	1691 (34.0)	20.0 ± 3.7	10.5 ± 3.7	9.5 ± 2.4	54.8 ± 8.5	6.4 ± 1.6
Medium	884 (34.8)	16.3 ± 4.0^2^	6.4 ± 3.2	9.8 ± 2.5^2^	43.7 ± 10.5^2^	4.2 ± 1.5^2^	1723 (34.6)	20.1 ± 3.7	11.1 ± 3.7^2^	9.0 ± 2.4^2^	52.4 ± 8.6^2^	6.8 ± 1.4^2^
High	825 (32.4)	16.2 ± 4.2^2^	6.5 ± 3.8	9.7 ± 2.6^2^	42.3 ± 10.6^2^	4.5 ± 1.6^2^	1561 (31.4)	20.4 ± 4.0^2^	11.8 ± 3.9^2^	8.6 ± 2.4^2^	51.2 ± 8.5^2^	7.1 ± 1.4^2^

Values are mean ± SD of the diet quality metrics.

1 AHEI-2010, Alternate Healthy Eating Index-2010; GDQS, Global Diet Quality Score; GDQS+, Global Diet Quality Score positive submetric; GDQS–, Global Diet Quality Score negative submetric; MDD-W, Minimum Dietary Diversity for Women; SES, socioeconomic status.

2 Different from reference group (*P* < 0.05).

We present correlation coefficients with energy-adjusted nutrient intakes in **Table 2**. The correlation observed in the 24-h recall data set between the diet quality metrics and the MPA was stronger for the GDQS than the MDD-W and the AHEI-2010. For the FFQ data, the GDQS and the MDD-W correlated in the same way with overall nutrient adequacy and outperformed the AHEI-2010. The GDQS was positively correlated with the intakes of calcium, folate, iron, vitamin A, vitamin B-12, zinc, fiber, and protein (rho = 0.09 to 0.38, *P* < 0.05) in both data sets and with total fat (rho = 0.03, *P* < 0.05) only using FFQ data; and was inversely correlated with the intake of added sugar in both data sets, and with total fat, SFA, and MUFA (rho = −0.06 to −0.16, *P* < 0.05) only using 24-h recall data. Compared with the MDD-W, the GDQS showed a stronger positive correlation with the intake of most micronutrients (except for vitamin A and vitamin B-12) and a stronger inverse correlation with the intake of added sugar. The MDD-W was not significantly correlated with the intake of total fat and SFA in the 24-h recall data and was positively correlated with these in the FFQ data. Compared with the AHEI-2010, the GDQS was more strongly correlated with the intake of all micronutrients and had a similar correlation with the intake of added sugar but a weaker correlation with fiber and all types of fat.

**TABLE 2:** Correlation between diet quality scores and nutrient intake in Mexican women^1^

	24-h recall (*n* = 2545)					FFQ (*n* = 4975)				
	GDQS	GDQS+	GDQS−	AHEI-2010	MDD-W	GDQS	GDQS+	GDQS−	AHEI-2010	MDD-W
Overall nutrient adequacy^2^	0.27^3^	0.16^3^	0.23^3^	0.14^3^	0.23 ^3^	0.37^3^	0.36^3^	0.04^4^	0.09^3^	0.37^3^
Calcium, mg/d	0.17^3^	0.02	0.24^3^	0.07^3^	0.17^3^	0.29^3^	0.14^3^	0.23^3^	0.05^4^	0.20^3^
Folate, μg/d	0.25^3^	0.24^3^	0.08^3^	0.25^3^	0.17^3^	0.33^3^	0.33^3^	0.01	0.25^3^	0.28^3^
Iron, mg/d	0.09^3^	0.09^3^	0.03	0.10^3^	0.08^3^	0.21^3^	0.16^3^	0.07^3^	0.15^3^	0.16^3^
Vitamin A, μg RAE/d	0.17^3^	0.15^3^	0.08^3^	0.05^4^	0.29^3^	0.29^3^	0.31^3^	−0.03^4^	0.16^3^	0.35^3^
Vitamin B-12, μg/d	0.05^4^	−0.04 ^4^	0.12^3^	−0.23^3^	0.18^3^	0.11^3^	0.11^3^	0.01	−0.19^3^	0.22^3^
Zinc, mg/d	0.24^3^	0.10^3^	0.24^3^	0.03	0.16^3^	0.29^3^	0.18^3^	0.17^3^	0.05^4^	0.22^3^
Fiber, g/d	0.38^3^	0.31^3^	0.21^3^	0.49^3^	0.17^3^	0.34^3^	0.20^3^	0.22^3^	0.56^3^	0.07^3^
Protein, g/d	0.29^3^	0.20^3^	0.21^3^	0.04	0.21^3^	0.35^3^	0.31^3^	0.07^3^	−0.05^4^	0.33^3^
MUFA, g/d	−0.14^3^	−0.07^3^	−0.13^3^	−0.25^3^	−0.01	−0.03	0.08^3^	−0.16^3^	−0.20^3^	0.12^3^
PUFA, g/d	0.17^3^	0.26^3^	−0.07^3^	0.23^3^	0.09^3^	0.01	0.09^3^	−0.14^3^	0.23^3^	0.03
Total fat, g/d	−0.06^4^	−0.05^4^	−0.03	−0.18^3^	0.04	0.03^4^	0.11^3^	−0.12^3^	−0.12^3^	0.14^3^
SFA, g/d	−0.16^3^	−0.21^3^	0.00	−0.30^3^	0.01	0.00	0.01	−0.02	−0.24^3^	0.11^3^
Added sugar, g/d	−0.37^3^	−0.17^3^	−0.38^3^	−0.36^3^	−0.09^3^	−0.38^3^	−0.21^3^	−0.28^3^	−0.40^3^	−0.13^3^

Values are Spearman correlation coefficients between the dietary scores and the energy-adjusted intake of nutrients.

1 AHEI-2010, Alternate Healthy Eating Index-2010; GDQS, Global Diet Quality Score; GDQS+, Global Diet Quality Score positive submetric; GDQS–, Global Diet Quality Score negative submetric; MDD-W, Minimum Dietary Diversity for Women; RAE, retinol activity equivalents.

2 Summary measure for the intake of calcium, fiber, folate, iron, protein, vitamin A, vitamin B-12, and zinc.

3 Spearman's *P* value < 0.001.

4 Spearman's *P* value < 0.05.

We present covariate-adjusted associations between diet quality scores and health parameters in **Table 3**. An increase of 1 SD of the GDQS was inversely associated with serum ferritin (β: −2.84 μg/L; 95% CI: −5.52, −0.17 μg/L), BMI (β: −0.25 kg/m^2^; 95% CI: −0.41, −0.08 kg/m^2^), WC (β: −0.81 cm; 95% CI: −1.31, −0.31 cm), total serum cholesterol (β: −2.91 mg/dL; 95% CI: −4.65, −1.16 mg/dL), and LDL cholesterol (β: −1.29 mg/dL; 95% CI: −2.46, −0.12 mg/dL) using FFQ data, and was positively associated with serum folate (β: 0.22 ng/mL; 95% CI: 0.01, 0.43 ng/mL) using 24-h recall data. The GDQS+ was inversely associated with serum ferritin, BMI, WC, and total serum cholesterol only in the FFQ data; whereas the GDQS– was not associated with any evaluated health parameter. The AHEI-2010 was inversely associated with BMI (β: −0.28; 95% CI: −0.45, −0.10) and WC (β: −0.68 cm; 95% CI: −1.22, −0.14 cm), and had a positive association with serum triglycerides (β: 5.03 mg/dL; 95% CI: 0.14, 9.93 mg/dL) only using FFQ data. The MDD-W had a positive association with serum folate (β: 0.37 ng/mL; 95% CI: 0.16, 0.58 ng/mL), LDL cholesterol (β: 2.09 mg/dL; 95% CI: 0.60, 3.57 mg/dL), HDL cholesterol (β: 0.73 mg/dL; 95% CI: 0.23, 1.23 mg/dL), and total cholesterol (β: 2.28 mg/dL; 95% CI: 0.41, 4.15 mg/dL) in the 24-h data set and was inversely associated with insulin concentrations (β: −0.45 μU/mL; 95% CI: −0.84, −0.06 μU/mL) in the FFQ data set.

**TABLE 3:** Association between diet quality scores and health markers in Mexican women^1^

	24-h recall					FFQ				
	GDQS	GDQS+	GDQS−	AHEI-2010	MDD-W	GDQS	GDQS+	GDQS−	AHEI-2010	MDD-W
Serum ferritin, μg/L	0.80 (−3.54, 1.93)	−0.10 (−2.73, 2.54)	−1.19 (−3.95, 1.57)	−0.19 (−2.99, 2.61)	−0.10 (−2.58, 2.78)	−2.84 (−5.52, −0.17)^2^	−3.42 (−6.07, −0.76)^2^	0.96 (−1.69, 3.62)	−1.72 (−4.74, 1.30)	−3.39 (−5.99, −0.79)^2^
Serum folate, ng/mL	0.22 (0.01, 0.43)^2^	0.20 (−0.01, 0.41)	0.08 (−0.13, 0.29)	0.02 (−0.20, 0.24)	0.37 (0.16, 0.58)^2^	−0.18 (−0.40, 0.03)	−0.06 (−0.28, 0.15)	−0.18 (−0.39, 0.03)	−0.12 (−0.37, 0.14)	−0.09 (−0.30, 0.13)
MetS components	−0.05 (−0.10, 0.01)	−0.04 (−0.09, 0.01)	−0.02 (−0.08, 0.04)	−0.03 (−0.08, 0.03)	−0.01 (−0.06, 0.04)	−0.03 (−0.07, 0.01)	−0.03 (−0.07, 0.01)	−0.01 (−0.04, 0.04)	−0.03 (−0.07, 0.01)	−0.02 (−0.06, 0.02)
BMI, kg/m^2^	−0.06 (−0.28, 0.16)	−0.10 (−0.32, 0.11)	0.04 (−0.17, 0.26)	−0.10 (−0.32, 0.12)	−0.03 (−0.25, 0.18)	−0.25 (−0.41, −0.08)^2^	−0.19 (−0.35, −0.03)^2^	−0.09 (−0.25, 0.07)	−0.28 (−0.45, −0.10)^2^	−0.09 (−0.25, 0.08)
Waist circumference, cm	−0.19 (−0.97, 0.59)	−0.13 (−0.88, 0.62)	−0.13 (−0.93, 0.66)	−0.57 (−1.36, 0.22)	0.24 (−0.50, 0.99)	−0.81 (−1.31, −0.31)^2^	−0.67 (−1.17, −0.17)^2^	−0.22 (−0.74, 0.29)	−0.68 (−1.22, −0.14)^2^	−0.50 (−1.01, 0.01)
Total cholesterol, mg/dL	0.98 (−0.95, 2.90)	1.30 (−0.58, 3.18)	−0.21 (−2.15, 1.73)	0.37 (−1.60, 2.35)	2.28 (0.41, 4.15)^2^	−2.91 (−4.65, −1.16)^2^	−2.17 (−3.93, −0.41)^2^	−1.39 (−3.25, 0.47)	−1.22 (−3.05, 0.60)	−1.73 (−3.56, 0.10)
LDL-C, mg/dL	0.65 (−0.88, 2.18)	1.15 (−0.34, 2.65)	−0.55 (−2.09, 0.99)	0.46 (−1.11, 2.02)	2.09 (0.60, 3.57)^2^	−1.29 (−2.46, −0.12)^2^	−0.82 (−1.99, 0.35)	−0.73 (−1.91, 0.44)	−0.89 (−2.12, 0.34)	−0.44 (−1.61, 0.73)
HDL-C, mg/dL	0.32 (−0.20, 0.83)	0.49 (−0.01, 0.99)	−0.16 (−0.68, 0.36)	−0.40 (−0.93, 0.13)	0.73 (0.23, 1.23)^2^	−0.26 (−0.64, 0.13)	−0.09 (−0.47, 0.29)	−0.27 (−0.65, 0.12)	−0.10 (−0.51, 0.31)	−0.29 (−0.67, 0.10)
Triglycerides, mg/dL	0.70 (−4.97, 6.38)	−1.60 (−7.14, 3.94)	3.42 (−2.30, 9.13)	2.07 (−3.75, 7.89)	−1.97 (−7.49, 3.54)	1.53 (−2.97, 6.03)	0.71 (−3.78, 5.20)	1.29 (−3.23, 5.81)	5.03 (0.14, 9.93)^2^	−0.27 (−4.76, 4.22)
Insulin, μU/mL	−0.16 (−0.66, 0.35)	0.16 (−0.33, 0.66)	−0.49 (−1.00, 0.02)	−0.18 (−0.70, 0.34)	0.06 (−0.43, 0.55)	−0.25 (−0.65, 0.14)	−0.37 (−0.76, 0.02)	0.19 (−0.20, 0.59)	−0.11 (−0.49, 0.28)	−0.45 (−0.84, −0.06)^2^
Glucose, mg/dL	−0.41 (−1.84, 1.01)	−0.59 (−1.98, 0.80)	0.15 (−1.28, 1.59)	−0.84 (−2.31, 0.62)	0.43 (−0.96, 1.81)	0.51 (−0.82, 1.83)	0.12 (−1.20, 1.44)	0.62 (−0.72, 1.95)	−0.56 (−2.0, 0.88)	0.08 (−1.24, 1.40)

Values are change [β (95% CI)] per 1 SD of the diet quality scores adjusted by age, area of residence (urban/rural), and SES.

1 AHEI-2010, Alternate Healthy Eating Index-2010; GDQS, Global Diet Quality Score; GDQS+, Global Diet Quality Score positive submetric; GDQS−, Global Diet Quality Score negative submetric; MDD-W, Minimum Dietary Diversity for Women; MetS, metabolic syndrome; SES, socioeconomic status.

2 *t*-Test *P* value  < 0.05. Original to this manuscript.

**FIGURE 1:**
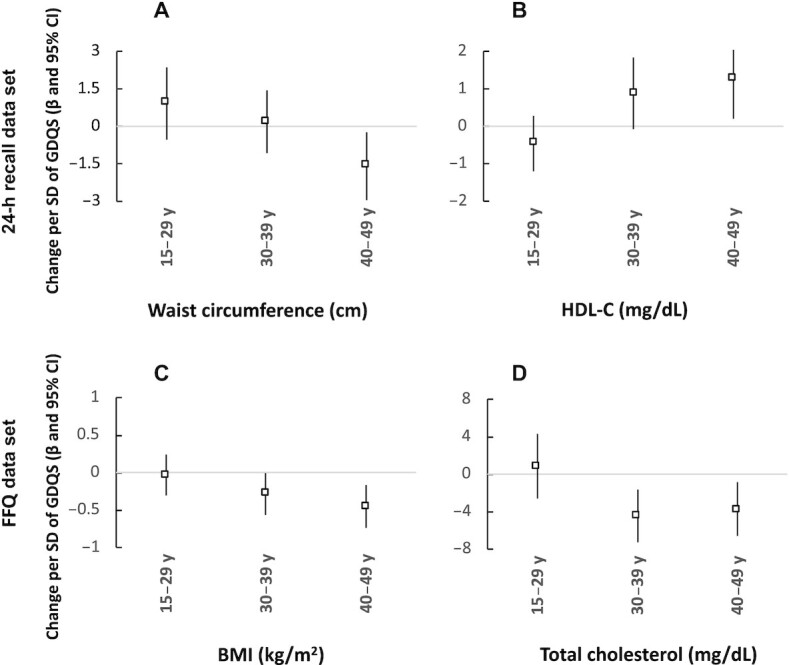
Association between the Global Diet Quality Score and health markers by age groups in Mexican women. Values are change [β (95% CI)] in (A) waist circumference, (B) serum HDL-C, (C) BMI, and (D) total serum cholesterol per 1 SD of the GDQS from models with a significative interaction term for age groups (*P* value < 0.10) and adjusted by age, area of residence (urban/rural), and socioeconomic status. GDQS, Global Diet Quality Score.

We present the significant interactions across age groups for the GDQS in **Figure 1**, for the AHEI-2010 in **Supplemental Figure 1**, and for the MDD-W in **Supplemental Figure 2**. There was a significant interaction between the GDQS and age groups for WC and HDL cholesterol in the 24-h recall data set, and for BMI and serum cholesterol in the FFQ data set (*P* for interaction < 0.10). The associations between the GDQS and WC, BMI, and HDL cholesterol were statistically significant only in women aged 40–49 y, and with serum cholesterol only in women aged ≥30 y. The AHEI-2010 showed a significant interaction between age groups for its association with BMI and WC in both data sets, with HDL cholesterol in the 24-h recall, and with LDL cholesterol and MetS in the FFQ data, with a significant inverse association with HDL cholesterol in women aged 15–29 y and with BMI and WC in women aged 30 y and above (Supplemental Figure 1). The MDD-W had a significant interaction between age groups for its association with BMI and LDL cholesterol for the 24-h recall data, and with serum cholesterol, HDL cholesterol, and triglycerides for the FFQ data, with a significant inverse association with total cholesterol and triglycerides only in women 30–39 y, and with HDL cholesterol only in women 40–49 y, and a positive association with LDL cholesterol in women 30–39 y (Supplemental Figure 2).

## Discussion

We used dietary data from 2 national Mexican surveys to evaluate the performance of the GDQS with nutrition and health outcomes in NPNL women of reproductive age. We found that the GDQS was associated with the intake of key nutrients relevant for their public health implications in LMICs and was also associated with health parameters related to the risk of NCDs (29–32).

The GDQS was positively correlated with nutrient adequacy and, with the exception of fat intake in the FFQ data set, correlated with the intake of nutrients involved in the development of NCDs. The weak correlation between the GDQS and the intake of all types of fats observed in the FFQ data set could be the result of difficulty in disaggregating individual foods from the items included in the questionnaire. For this analysis, we used standard recipes to disaggregate complex dishes but did not separate individual foods from simple preparations (i.e. fried beans and fried eggs, which were counted in the groups of legumes and eggs, respectively), resulting in healthy components of the GDQS positively correlated with fat intake. Moreover, the nutritional composition assigned to the food items in the FFQ does not account for the variation in the nutritional profile of the individual foods that would normally differ between individuals; therefore, the detailed information provided in the 24-h recall allows for a more accurate estimation of individual nutrient intake (11, 33). In the case of MUFA, we believe the unexpected results had to do with the low intake of nuts and seeds and liquid oils with a high content of MUFA in our population. Hence, red and processed meat become an important source of MUFA.

The GDQS and the MDD-W showed a comparable correlation with overall nutrient adequacy but the GDQS was more strongly correlated with the intake of added sugar and fiber, and using 24-h recall data was inversely correlated with the intake of total fat and SFA, whereas the MDD-W had a positive correlation with these fats in both data sets. The GDQS and MDD-W were positively associated with serum folate concentrations only in the 24-h recall data set. Evidence suggests that serum folate can take weeks to respond to increased intake (34, 35), which contradicts the association found with the 24-h recall and the lack of association observed using an FFQ. However, our results are consistent with other studies that have found a stronger correlation between folate intake and serum folate when assessing diet using a 24-h recall compared with an FFQ (36). This could be explained because the 24-h recall is better suited to reflect nutrient intake for its ability to capture a wide range of foods and get more precise quantity estimates, whereas the FFQ is limited by the amount of food items included in the instrument.

The positive associations observed between the MDD-W and serum cholesterol, HDL cholesterol, and LDL cholesterol in the 24-h recall data set could be explained because, as an indicator of dietary diversity, the MDD-W might reflect an increased intake of unhealthy foods as well as healthy foods, which is also consistent with its positive correlation with the intake of total fat and SFA (although only statistically significant in the FFQ data). The AHEI-2010 showed a stronger correlation than the GDQS with the intake of fiber and all types of fat but had a low or inverse correlation with the intake of micronutrients and was associated with fewer health parameters than the GDQS.

In the evaluation of interaction by age groups, we found a significant association between the GDQS and BMI, WC, serum HDL cholesterol, and total cholesterol only among older women. In the same way, the AHEI-2010 was inversely associated with BMI and WC only among women aged 30 y and above. Studies that have evaluated the influence of age on the response of blood lipids and body weight to dietary exposures show mixed results, with very few focused on the differences among women of reproductive age (37–42). However, some studies have found a stronger effect of dietary interventions on plasma cholesterol, LDL cholesterol, and on weight loss among older compared with younger individuals (40–42), which could be explained by the age-related deterioration of the biological mechanisms that help mitigate the deleterious effects of diet, particularly on lipid metabolism (40, 43).

The overall performance of the GDQS to measure both dimensions of diet quality was better than the performance of the GDQS+ and GDQS– submetrics. The correlation of the submetrics with nutrient intake was generally weaker compared with the GDQS and both were associated with fewer health parameters. Interestingly, the GDQS− showed a stronger correlation than the GDQS+ with overall nutrient adequacy, vitamin B-12, and zinc using 24-h recall data and with calcium in both data sets. This could be explained because the high fat dairy and red meat groups in the GDQS– are scored in a nonlinear way, increasing its score as intake increases and receiving low scores only with very high or very low intakes. In the case of high fat dairy, <2% of women consume very high intakes (data not shown) and therefore, this group would be for the most part positively correlated with the intake of nutrients provided by this food group. These results also highlight the role of these food groups to contribute to micronutrient intake in women from resource-limited countries such as Mexico.

The mean values for the GDQS+ and the GDQS− submetrics were higher among older women compared with their younger counterparts, following a pattern that was consistent across data sets for all evaluated metrics. The GDQS− and the AHEI-2010 were distributed in the same way across urban/rural area and SES. In contrast, the distribution of the GDQS+ among subpopulations differed across dietary instruments, following the same pattern as the MDD-W only for the FFQ data. Even though the distribution of the GDQS submetrics across subpopulations showed similarities to the AHEI-2010 and the MDD-W that are consistent with their intended purpose to reflect each aspect of dietary quality, the submetrics offered the advantage of providing useful information for the characterization of the intake of healthy and unhealthy components in different population groups. For instance, dietary quality improved with age as a result of higher scores for the intake of both healthy (GDQS+) and unhealthy (GDQS−) foods. In the comparison across rural/urban area and SES, the submetrics’ distributions suggest that the consumption of unhealthy foods is lower among women from more vulnerable backgrounds but the intake of healthy foods seems to be more heterogeneous. Previous studies in Mexico have shown that those from rural areas and low SES consume less fruits and vegetables, but more legumes and whole grains (44, 45). Furthermore, it has been documented that in comparison to the 24-h recall, the FFQ overestimates the intake of fruits and vegetables (46). Altogether, this could explain the inconsistent findings in the GDQS+ scores by rural/urban area and SES across dietary instruments.

The 24-h recall and the FFQ both showed an overall good performance of the GDQS but reflected the 2 dimensions of diet quality in a slightly different way. For nutrient adequacy, the GDQS showed a significant correlation with micronutrient intake when using both instruments but was positively associated with serum folate concentrations only using 24-h recall data. Conversely, the GDQS was associated with anthropometric and biochemical markers of NCDs only using FFQ data but had a more consistent correlation with the intake of all types of fats (except for MUFA) in relation to their risk of NCDs using 24-h recall data. The stronger associations observed between the GDQS and markers of chronic disease in the FFQ data compared with the 24-h recall data could be explained by the advantage of the FFQ to better reflect long-term intake (47, 48). However, even though we did not find a significant association between the GDQS and markers of chronic disease in the overall sample with 24-h recall data, the interaction analysis found a significant association of the GDQS with WC and HDL cholesterol in women aged 40 to 49 y. Furthermore, we computed the GDQS and other metrics using information from a single 24-h recall and therefore, estimates of association are expected to be attenuated because of the measurement error from the within-person variation of food intake that has not been accounted for in the metric estimation. This was done in order to test the performance of the GDQS following a simple methodology that would fit the limited data available in low-resources settings.

This study has several strengths. First, we conducted a pooled analysis of 2 national surveys that provide robust data for the assessment of diet quality compared with multiple health parameters and for the comparisons across different population groups. Second, we evaluated the performance of the GDQS using dietary data obtained with 2 different instruments and thus allows for a better understanding of the advantages and disadvantages provided by both instruments for this purpose. Third, the 2012 and 2016 surveys collected information over separate time periods that together reflect food intake across all seasons. Finally, the subsample with a second 24-h recall provided information to correct for the within-person variation of nutrient intake to obtain estimates for usual intake.

The main limitation of this study is the use of cross-sectional data which does not permit the evaluation of the causal association between diet quality and health outcomes. Measuring both exposure and outcome at a single timepoint limits the ability to elucidate the nature of the observed association, including the possibility of reverse causality. This could be partially mitigated by the use of an instrument such as the FFQ. Moreover, self-reported intake is susceptible to measurement error and it is well known that energy intake is differentially misreported according to factors such as BMI and education (49–52). Mexico is a country with a high burden of obesity (4), a factor known to be associated with underreporting energy intake; making dietary data obtained from our population more prone to error than others. Nevertheless, a clear association was observed between the diet quality metrics with nutrient intake and health outcomes that is consistent with what we expected given the characteristics of each metric. It is important to note that we have evaluated the performance of the GDQS using data collected with validated instruments that collect detailed information and may not reflect the performance of the GDQS when using the GDQS app, which was designed to provide a low-cost and easy-to-use alternative when more robust instruments are not available but may also provide less precise estimates, although this should be evaluated first. Lastly, results presented here are limited to NPNL women of reproductive age and therefore, it is unclear if our findings would be applicable to other demographic groups.

In conclusion, the GDQS was associated with the intake of key nutrients and with health parameters related to the risk of chronic disease in Mexican women of reproductive age. The GDQS is a simple metric that is easy to tabulate and does not require a high input of data, such as food composition tables. In Mexican NPNL women of reproductive age it was robust enough to reflect the 2 main dimensions of diet quality that are usually evaluated separately, with a performance comparable to the MDD-W to evaluate nutrient adequacy and to the AHEI-2010 to reflect risk of chronic disease when using dietary data collected with a 24-h recall or a past-week FFQ. Both instruments for data collection were useful to evaluate the performance of the GDQS, but the 24-h recall seems to be more appropriate for population-level descriptive studies for its ability to capture absolute nutrient intake, whereas the FFQ may be more appropriate for analytical studies that prioritize the evaluation of long-term intake. The submetrics derived from the GDQS (GDQS+ and GDQS–) had an overall performance inferior to the GDQS but were helpful for the characterization across subpopulations of the intake of healthy and unhealthy dietary components in relation to overall dietary quality, which can provide useful information for targeting interventions. Further evaluations should include the evaluation of the GDQS in other demographic groups and the use of longitudinal data to reduce the risk of bias and provide a better understanding of the nature of these associations.

## Supplementary Material

nxab202_Supplemental_Files

## Notes

Funding for the research was provided by FHI Solutions, recipient of a Bill and Melinda Gates Foundation grant to support Intake – Center for Dietary Assessment.

Published in a supplement to the Journal of Nutrition. Publication costs for this supplement were funded by the Bill & Melinda Gates Foundation in conjunction with FHI Solutions, recipient of a Bill & Melinda Gates Foundation grant to support Intake – Center for Dietary Assessment. The opinions expressed in this publication are those of the authors and are not attributable to the sponsors or the publisher, Editor, or Editorial Board of *The Journal of Nutrition*.

The Supplement Coordinators for the supplement publication were Megan Deitchler, Intake – Center for Dietary Assessment at FHI Solutions, Washington, DC; and Sabri Bromage, Harvard T.H. Chan School of Public Health, Boston, MA.

Author disclosures: TTF is an Associate Editor for the Journal of Nutrition and played no role in the Journal’s evaluation of the manuscript. All other authors report no conflicts of interest.

Supplemental Table 1 and Supplemental Figures 1 and 2 are available from the “Supplementary data” link in the online posting of the article and from the same link in the online table of contents at https://academic.oup.com/jn/.

The GDQS research initiative was launched by Intake – Center for Dietary Assessment. The research was led by Harvard T.H. Chan School of Public Health, Department of Nutrition and carried out in collaboration with researchers at the National Public Health Institute (INSP), Mexico. Funding for the research was provided by FHI Solutions, recipient of a Bill & Melinda Gates Foundation grant to support Intake – Center for Dietary Assessment.

Abbreviations used: AHEI-2010, Alternate Healthy Eating Index-2010; BLUP, best linear unbiased predictor; EAR, estimated average requirement; ENSANUT, Mexican National Health and Nutrition Survey; GDQS, Global Diet Quality Score; GDQS+, Global Diet Quality Score positive submetric; GDQS–, Global Diet Quality Score negative submetric; INSP, Mexican National Institute of Public Health; LMIC, low- and middle-income country; MDD-W, Minimum Dietary Diversity for Women; MetS, metabolic syndrome; MPA, mean probability of adequacy; NCD, noncommunicable disease; NPNL, nonpregnant nonlactating; SES, socioeconomic status; WC, waist circumference.
