# Supplementary material for: Performance of the Global Diet Quality Score with Nutrition and Health Outcomes in Mexico with 24-h Recall and FFQ Data
Source: J Nutr. 2021 Oct 23;151(Suppl 2):143S–151S. doi: 10.1093/jn/nxab202 (PMC8542100; doi:10.1093/jn/nxab202)
Supplement: nxab202_Supplemental_Files [file nxab202_supplemental_files.zip › Supplemental data_Figure 1.pdf]

Supplemental Figure 1. Association between the AHEI-2010 and health markers by age groups in Mexican women

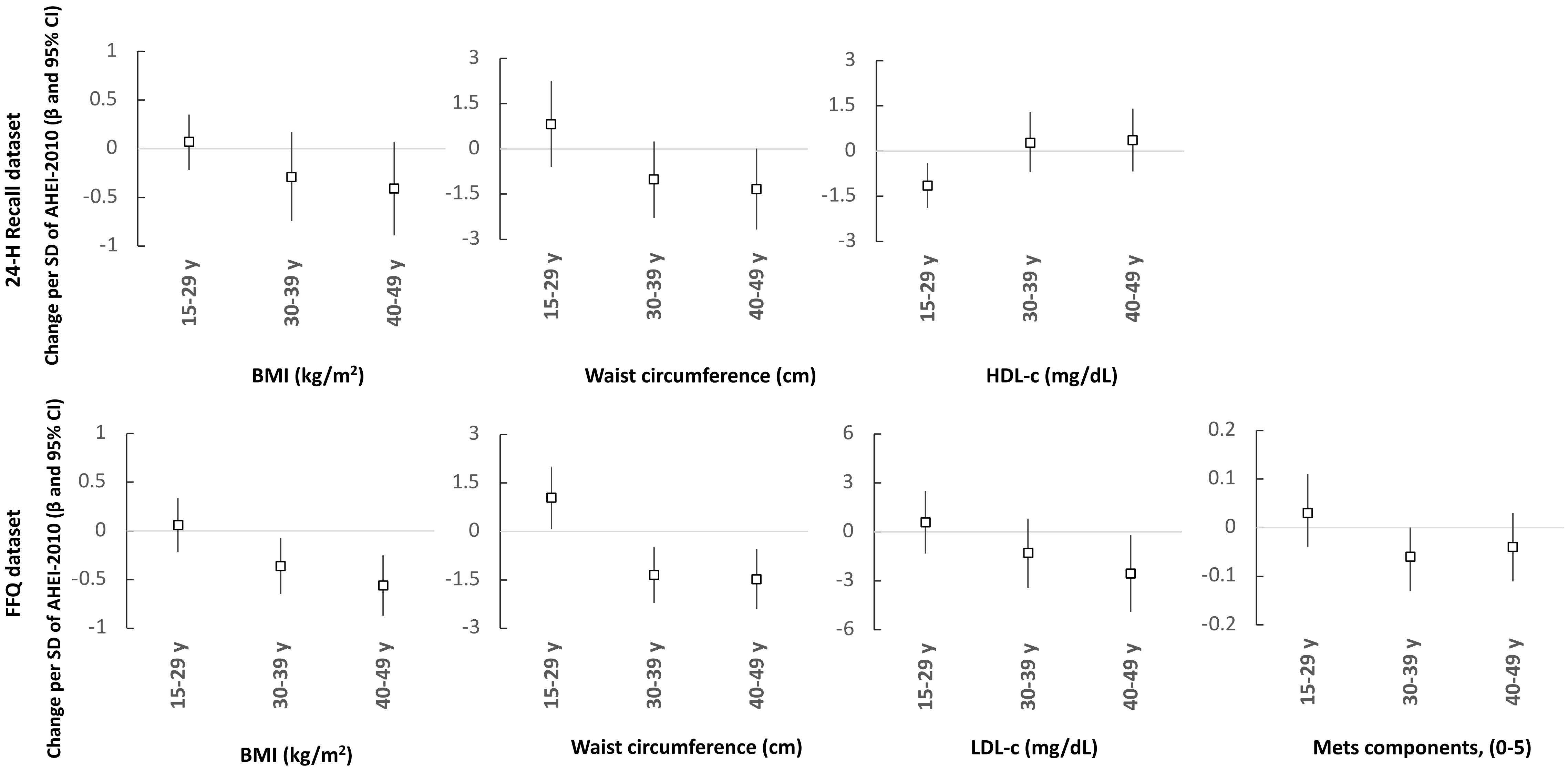

Values are change [β (95% CI)] per 1 SD of the AHEI-2010 from models with a significative interaction term for age groups (p-value <0.10) and adjusted by age, area of residence (urban/rural), and socioeconomic status..
AHEI-2010: Alternate Healthy Eating Index-2010; BMI: Body mass index; FFQ: Food frequency questionnaire; HDL-c: High-density lipoprotein cholesterol; LDL-c: High-density lipoprotein cholesterol; MetS: Metabolic syndrome
