# Supplementary material for: Performance of the Global Diet Quality Score with Nutrition and Health Outcomes in Mexico with 24-h Recall and FFQ Data
Source: J Nutr. 2021 Oct 23;151(Suppl 2):143S–151S. doi: 10.1093/jn/nxab202 (PMC8542100; doi:10.1093/jn/nxab202)
Supplement: nxab202_Supplemental_Files [file nxab202_supplemental_files.zip › Supplemental data_Table 1.pdf]

Supplemental table 1. Distribution of the food groups included in the Global Diet Quality Score by sociodemographic characteristics

			24-H Recall								
			All	Age			Area of residence		Socioeconomic status		
				15-29 y	30-39 y	40-49 y	Rural	Urban	Low	Medium	High
			N=2542	N=1411	N=621	N=510	N=1030	N=1512	N=833	N=884	N=825
**Healthy food groups**	Min.	Max.									
Whole grains	0	2	1.3 (1.0)	1.3 (1.0)	1.3 (0.9)	1.3 (0.9)	1.4 (0.9)	1.2 (1.0)	1.4 (0.9)	1.2 (1.0)	1.2 (1.0)
Citrus fruits	0	2	0.2 (0.5)	0.2 (0.5)	0.2 (0.5)	0.2 (0.6)	0.2 (0.5)	0.2 (0.6)	0.2 (0.5)	0.2 (0.5)	0.2 (0.6)
Deep orange fruits	0	2	0.2 (0.5)	0.2 (0.5)	0.2 (0.5)	0.2 (0.6)	0.2 (0.6)	0.2 (0.5)	0.2 (0.5)	0.2 (0.5)	0.2 (0.5)
Other fruits	0	2	0.5 (0.8)	0.5 (0.8)	0.5 (0.8)	0.6 (0.8)	0.5 (0.8)	0.5 (0.8)	0.4 (0.7)	0.5 (0.8)	0.6 (0.9)
Cruciferous vegetables	0	0.5	0.0 (0.1)	0.0 (0.1)	0.0 (0.1)	0.0 (0.1)	0.0 (0.1)	0.0 (0.1)	0.0 (0.1)	0.0 (0.1)	0.0 (0.1)
Dark green leafy vegetables	0	4	0.3 (1.0)	0.3 (1.0)	0.4 (1.0)	0.4 (1.0)	0.3 (0.9)	0.4 (1.0)	0.3 (0.9)	0.3 (1.0)	0.4 (1.1)
Deep orange vegetables	0	0.5	0.0 (0.1)	0.0 (0.1)	0.0 (0.1)	0.0 (0.1)	0.0 (0.1)	0.0 (0.1)	0.0 (0.1)	0.0 (0.1)	0.0 (0.1)
Other vegetables	0	0.5	0.3 (0.2)	0.3 (0.2)	0.3 (0.2)	0.3 (0.2)	0.3 (0.2)	0.3 (0.2)	0.3 (0.2)	0.3 (0.2)	0.3 (0.2)
Deep orange tubers	0	0.5	0.0 (0.0)	0.0 (0.0)	0.0 (0.0)	0.0 (0.0)	0.0 (0.0)	0.0 (0.0)	0.0 (0.0)	0.0 (0.0)	0.0 (0.0)
Legumes	0	4	1.1 (1.5)	1.0 (1.4)	1.2 (1.5)	1.3 (1.6)	1.3 (1.6)	0.9 (1.4)	1.4 (1.6)	1.0 (1.4)	0.8 (1.4)
Nuts and seeds	0	4	0.1 (0.7)	0.1 (0.6)	0.2 (0.8)	0.2 (0.8)	0.2 (0.8)	0.1 (0.6)	0.1 (0.7)	0.1 (0.7)	0.1 (0.7)
Liquid oils	0	2	1.0 (0.8)	1.1 (0.8)	1.0 (0.8)	1.1 (0.8)	1.0 (0.8)	1.1 (0.8)	1.0 (0.8)	1.1 (0.8)	1.0 (0.8)
Poultry	0	2	0.5 (0.8)	0.5 (0.8)	0.5 (0.8)	0.5 (0.8)	0.5 (0.8)	0.5 (0.8)	0.4 (0.8)	0.5 (0.8)	0.6 (0.8)
Eggs	0	2	0.7 (0.9)	0.6 (0.9)	0.7 (0.9)	0.7 (0.9)	0.7 (0.9)	0.7 (0.9)	0.7 (0.9)	0.7 (0.9)	0.6 (0.9)
Fish	0	2	0.1 (0.4)	0.1 (0.4)	0.1 (0.4)	0.2 (0.5)	0.1 (0.5)	0.1 (0.4)	0.1 (0.4)	0.1 (0.5)	0.1 (0.5)
Low fat dairy	0	2	0.1 (0.4)	0.1 (0.4)	0.1 (0.4)	0.1 (0.4)	0.1 (0.4)	0.1 (0.5)	0.1 (0.3)	0.1 (0.4)	0.2 (0.5)
**Unhealthy food groups**											
Refined grains and baked goods	0	2	0.4 (0.8)	0.4 (0.8)	0.4 (0.8)	0.4 (0.8)	0.5 (0.8)	0.4 (0.7)	0.6 (0.8)	0.4 (0.7)	0.3 (0.7)
White roots and tubers	0	2	1.7 (0.7)	1.6 (0.7)	1.7 (0.6)	1.7 (0.6)	1.7 (0.6)	1.6 (0.7)	1.7 (0.6)	1.6 (0.7)	1.6 (0.7)
Processed meat	0	2	1.6 (0.7)	1.6 (0.8)	1.7 (0.7)	1.7 (0.7)	1.7 (0.7)	1.5 (0.8)	1.7 (0.7)	1.6 (0.7)	1.5 (0.8)
Red meat	0	1	0.1 (0.3)	0.1 (0.3)	0.1 (0.3)	0.1 (0.3)	0.1 (0.3)	0.2 (0.4)	0.1 (0.3)	0.1 (0.4)	0.2 (0.4)
High-fat dairy	0	2	0.8 (0.9)	0.8 (0.9)	0.8 (0.9)	0.7 (0.9)	0.7 (0.9)	0.9 (0.9)	0.7 (0.9)	0.8 (0.9)	0.9 (0.9)
Fried foods	0	2	1.5 (0.8)	1.4 (0.9)	1.6 (0.7)	1.7 (0.7)	1.6 (0.8)	1.5 (0.8)	1.6 (0.8)	1.5 (0.8)	1.5 (0.8)
Sweets and ice cream	0	2	1.2 (0.8)	1.2 (0.8)	1.2 (0.8)	1.3 (0.8)	1.3 (0.8)	1.2 (0.8)	1.3 (0.8)	1.2 (0.8)	1.2 (0.8)
Juice	0	2	1.4 (0.9)	1.4 (0.9)	1.4 (0.9)	1.4 (0.9)	1.5 (0.9)	1.3 (0.9)	1.5 (0.9)	1.4 (0.9)	1.3 (0.9)
Sugar-sweetened beverages	0	2	1.2 (1)	1.1 (1)	1.2 (0.9)	1.3 (0.9)	1.2 (0.9)	1.2 (1.0)	1.3 (0.9)	1.1 (1.0)	1.1 (1.0)

			Food Frequency Questionnaire								
			All	Age			Area of residence		Socioeconomic status		
				15-29 y	30-39 y	40-49 y	Rural	Urban	Low	Medium	High
			N=4975	N=1868	N=1661	N=1446	N=2209	N=2766	N=1691	N=1723	N=1561
**Healthy food groups**	Min.	Max.									
Whole grains	0	2	1.9 (0.5)	1.8 (0.5)	1.9 (0.5)	1.9 (0.4)	1.9 (0.5)	1.9 (0.5)	1.8 (0.5)	1.9 (0.5)	1.9 (0.4)
Citrus fruits	0	2	0.4 (0.7)	0.5 (0.7)	0.4 (0.7)	0.4 (0.7)	0.4 (0.7)	0.4 (0.7)	0.4 (0.7)	0.4 (0.7)	0.4 (0.7)
Deep orange fruits	0	2	0.4 (0.7)	0.4 (0.6)	0.4 (0.7)	0.5 (0.7)	0.4 (0.7)	0.4 (0.7)	0.4 (0.7)	0.4 (0.7)	0.5 (0.7)
Other fruits	0	2	1.1 (0.8)	1.0 (0.8)	1.1 (0.8)	1.2 (0.8)	1.1 (0.8)	1.1 (0.8)	1.0 (0.8)	1.1 (0.8)	1.3 (0.8)
Cruciferous vegetables	0	0.5	0.1 (0.1)	0.0 (0.1)	0.0 (0.1)	0.1 (0.1)	0.0 (0.1)	0.1 (0.1)	0.0 (0.1)	0.0 (0.1)	0.1 (0.1)
Dark green leafy vegetables	0	4	1.1 (1.4)	1.0 (1.3)	1.2 (1.4)	1.2 (1.5)	1.0 (1.4)	1.2 (1.4)	0.9 (1.3)	1.1 (1.4)	1.4 (1.5)
Deep orange vegetables	0	0.5	0.1 (0.2)	0.1 (0.1)	0.1 (0.2)	0.1 (0.2)	0.1 (0.1)	0.1 (0.2)	0.1 (0.1)	0.1 (0.1)	0.1 (0.2)
Other vegetables	0	0.5	0.3 (0.2)	0.3 (0.2)	0.3 (0.2)	0.3 (0.2)	0.3 (0.2)	0.3 (0.2)	0.3 (0.2)	0.3 (0.2)	0.4 (0.2)
Deep orange tubers	0	0.5	0.0 (0.0)	0.0 (0.0)	0.0 (0.0)	0.0 (0.0)	0.0 (0.0)	0.0 (0.0)	0.0 (0.0)	0.0 (0.0)	0.0 (0.0)
Legumes	0	4	3.1 (1.3)	2.9 (1.4)	3.1 (1.3)	3.2 (1.3)	3.3 (1.2)	2.9 (1.4)	3.2 (1.3)	3.1 (1.3)	2.8 (1.4)
Nuts and seeds	0	4	0.2 (0.7)	0.2 (0.7)	0.2 (0.7)	0.3 (0.8)	0.2 (0.7)	0.3 (0.7)	0.2 (0.6)	0.2 (0.7)	0.3 (0.8)
Liquid oils	0	2	0.5 (0.7)	0.5 (0.7)	0.5 (0.7)	0.5 (0.6)	0.5 (0.6)	0.5 (0.7)	0.4 (0.6)	0.5 (0.7)	0.5 (0.7)
Poultry	0	2	0.6 (0.7)	0.5 (0.7)	0.6 (0.7)	0.6 (0.7)	0.5 (0.6)	0.6 (0.7)	0.4 (0.6)	0.6 (0.7)	0.7 (0.7)
Eggs	0	2	1.1 (0.8)	1.1 (0.8)	1.2 (0.8)	1.1 (0.8)	1.1 (0.8)	1.1 (0.8)	1.1 (0.8)	1.1 (0.8)	1.1 (0.8)
Fish	0	2	0.2 (0.4)	0.2 (0.4)	0.2 (0.4)	0.2 (0.4)	0.1 (0.4)	0.2 (0.5)	0.1 (0.4)	0.2 (0.4)	0.2 (0.5)
Low fat dairy	0	2	0.1 (0.4)	0.1 (0.4)	0.1 (0.4)	0.2 (0.5)	0.1 (0.4)	0.2 (0.5)	0.1 (0.4)	0.1 (0.4)	0.2 (0.5)
**Unhealthy food groups**											
Refined grains and baked goods	0	2	0.1 (0.4)	0.1 (0.4)	0.1 (0.4)	0.1 (0.4)	0.2 (0.4)	0.1 (0.4)	0.2 (0.5)	0.1 (0.4)	0.1 (0.3)
White roots and tubers	0	2	1.5 (0.7)	1.5 (0.6)	1.5 (0.7)	1.5 (0.7)	1.5 (0.6)	1.5 (0.7)	1.5 (0.6)	1.5 (0.6)	1.4 (0.7)
Processed meat	0	2	1.4 (0.7)	1.3 (0.8)	1.4 (0.7)	1.5 (0.7)	1.5 (0.7)	1.3 (0.7)	1.5 (0.7)	1.3 (0.7)	1.3 (0.7)
Red meat	0	1	0.4 (0.5)	0.3 (0.5)	0.4 (0.5)	0.4 (0.5)	0.3 (0.5)	0.4 (0.5)	0.3 (0.4)	0.4 (0.5)	0.4 (0.5)
High-fat dairy	0	2	1.1 (0.8)	1.2 (0.8)	1.1 (0.8)	1.1 (0.8)	1.0 (0.8)	1.2 (0.8)	0.9 (0.8)	1.2 (0.8)	1.3 (0.8)
Fried foods	0	2	1.5 (0.8)	1.4 (0.8)	1.5 (0.8)	1.6 (0.7)	1.5 (0.7)	1.4 (0.8)	1.6 (0.7)	1.5 (0.8)	1.4 (0.8)
Sweets and ice cream	0	2	1.0 (0.8)	0.8 (0.8)	1.1 (0.8)	1.1 (0.8)	1.1 (0.8)	0.9 (0.8)	1.1 (0.8)	1.0 (0.8)	0.8 (0.8)
Juice	0	2	1.0 (0.9)	1.1 (0.9)	1.0 (0.9)	1.1 (0.9)	1.1 (0.9)	1.0 (0.9)	1.2 (0.9)	1.0 (0.9)	0.9 (0.9)
Sugar-sweetened beverages	0	2	1.1 (0.8)	0.9 (0.8)	1.1 (0.8)	1.2 (0.8)	1.1 (0.8)	1.0 (0.9)	1.2 (0.8)	1.0 (0.8)	1.0 (0.8)

Values are mean (SD). Data from non-pregnant non-lactating Mexican women of reproductive age from the National Health and Nutrition Survey (ENSANUT) 2012 and 2016.
